# Effects of methotrexate and etanercept treatment in moderate and severe psoriasis

**DOI:** 10.1097/MD.0000000000031527

**Published:** 2022-11-11

**Authors:** Elizabeth Benites, Esmeralda Carrillo, Martha Heras

**Affiliations:** a Catholic University of Santiago de Guayaquil: Universidad Catolica de Santiago de Guayaquil, Guayaquil, Ecuador; b Department of Human Anatomy and Embryology, Faculty of Medicine, Granada University, Granada, Spain; c Dermatology Physician, Hospital de Especialidaes “Dr. Teodoro Maldonado Carbo”, Guayaquil, Ecuador.

**Keywords:** BMI, etanercept, methotrexate, moderate and several psoriasis, overweight

## Abstract

Psoriasis is a disease of immunological origin that damages the skin and mucous membranes. Biological therapies with systemic medications are effective in treating moderate and severe psoriasis. Our objective was to evaluate the effects of methotrexate and etanercept treatment in this disease and to verify their response to the Psoriasis area and severity index (PASI) index in the initial and control phase. The number of patients treated at the Military Hospital in Guayaquil was 2.620 corresponding from July 2020 to July 2021; the selected sample according to the inclusion criteria was 94 patients with moderate and severe psoriasis. The method was retrospective, observational, descriptive, cross-sectional, correlational differential analytical, and approved by the Human Subjects Ethics Committee of the Specialties Hospital “Dr Teodoro Maldonado Carbo” of Guayaquil, Ecuador. In this study, the prevalence was 3.58%, and the body mass index was 28.13 corresponding to overweight and obesity. The PASI index in the initial stage before treatment was 10.8% and in the control phase, it decreased to 2.99%, showing a decrease in lesions and good improvement in the treatment of moderate and severe psoriasis. Student´s T, the combination of etanercept with methotrexate was compared with the response with the PASI index in the initial and control phases, presenting a value lower than 0.001, *P* = .05, which was very significant. In our study, treatment with etanercept and methotrexate in moderate and severe psoriasis had is a favorable response in reducing this disease. It is expected that, in Ecuador, the health authorities would implement the biologics for the treatment of moderate and several psoriasis and including them in the basic list of medicines of the Public Health Ministry.

## 1. Introduction

Psoriasis is a chronic cutaneous disease of immunological affectation that damages the skin, mucous membranes, scalp, and nails; with genetic predisposition, and can involve the joints. Environmental and psychosocial factors can aggravate the evolution of this disease which influences the quality and survival of these patients.^[1]^ This pathology affects between 3% of the population worldwide, presenting variations depending on age, gender, ethnicity, and geographical area, it is estimated that, in the world, there are 175 million affected with psoriasis, according to World Health Organization (WHO) 2019 the prevalence ranges between 1% and 3% of the world population. At the 67th World Health Assembly, held from May to 19–24, 2014, the WHO described psoriasis as “a chronic, non-contagious, painful, disfiguring, and disabling disease for which there is no cure, the etiology is unknown.^[2]^

The etiology of psoriasis is unknown; however, multiple studies have linked psoriasis to environmental, immunological, and genetic factors. It has also been linked to various histocompatibility antigens. The major susceptibility locus for psoriasis development of psoriasis is Psoriasis type one, which is located on chromosome 6p21 and is a risk factor for type I psoriasis.^[3]^

In Ecuador the prevalence of the disease is not known at the national level or by geographical areas, only reports of works of health units published in national journals, the information at the public level is general and this disease is not recorded with in the picture of autoimmune diseases although psoriasis is considered a multisystemic disease.^[4]^

Moderate and severe psoriasis known as Vulgaris is the most typical form of this disease that can occur in mild, moderate, and severe forms, whose origin is presented as the most frequent desquamative type, these are composed of dead cells that are detached in the form of plaque, commonly located on the chest, abdomen, knees, elbow, scalp, hands and feet, presenting the Auspitz sign in most cases. At the beginning of the clinical picture, these lesions appear with redness, inflammation, desquamation, pain, itching, and cracking, and the diagnosis is made by observing, We *evaluated* all methods used previously, the area of the lesion according to its intensity and extension, many times, it is necessary to perform biopsies to confirm the diagnosis ruling out other pathologies that may share the appearance and associated symptoms.^[5]^

Due to the good response of drugs that act on T lymphocytes in their pro-inflammatory receptors, this disease is associated with other pathologies (comorbidities) endocrine, metabolic, osteoarticular, and psychosocial factors that aggravate the quality of life in this type of patient.^[6]^

In 2018, a study conducted by the Ecuadorian Psoriasis Foundation determined that approximate 0.59% of the country’s population suffers from this condition, in addition to a marked trend toward an increase in cases; currently, there are approximately 100,000 Ecuadorians diagnosed with psoriasis, with a predilection for male patients, between 40 and 60 years of age.^[7]^

Psoriasis can be classified according to its type, pathogenesis, and severity for the latter, the Psoriasis area and severity index (PASI) scores scale assesses the severity of the lesions together with the percentage of body area affected.^[8]^

As therapeutic strategies to manage this condition, we will use topical drugs such as emollients, corticosteroids, and vitamin D analogs, such as calcipotriol methotrexate, which are anti-inflammatory drug developed as analog of folic acid.^[9]^ However, these alternatives only relieve the cutaneous symptoms of psoriasis without affecting the underlying causes of this dermatological condition. Despite this, it is also necessary to have optimal family and psychological support to ensure the effectiveness of all treatment strategies employed, in addition to making lifestyle changes to avoid comorbidities such as overweight and obesity, among others.^[10]^

Currently, the use of biological agents that seek to attenuate the inflammatory cascade that triggers psoriasis is a highly effective alternative for the management of this pathology, since, in this way, 1 of the main underlying causes of the disease is modified, improving the quality of life of patients.^[11]^

A study conducted with 1187 patients treated with 50 or 100 mg per week of etanercept reported a better response in patients with normal weight.^[12]^

In this study, most of the patients received treatment with topical and oral urea, topical calcipotriol, local betamethasone, loratadine tablets, methotrexate, and etanercept. For this reason, our objective was to test the response to treatments with etanercept and methotrexate in patients with moderate and severe psoriasis, the pathogenic basis body mass index (BMI) (muscle mass index) and PASI evaluation at the beginning and control of its administration.

## 2. Materials and methods

The data universe was 2820 active and passive duty military patients and their families who were treated at the Military Hospital of the city of Guayaquil from July 2020 to July 2021. Of these 262 had various skin diseases and annexes, of which 94 patients with a confirmatory diagnosis of moderate psoriasis: belonging to the outpatient clinic of the Department of Dermatology of the hospital. This work was non-experimental, epidemiology, retrospective, cross-sectional, observational, descriptive, correlational and differential analytical study, approved by the Human Subjects Ethics Committee of the Specialties Hospital “Dr Teodoro Maldonado Carbo” of Guayaquil, Ecuador.

The database was obtained from the electronic medical records of each patient at the military hospital. Data were analyzed using SPSS and Excel. The treatments used for moderate and severe psoriasis were topical, oral drugs, injectable, and biologics.

The 94 patients, all adults confirmed with moderate and severe psoriasis, received the following treatment: initial dose of methotrexate 5 mg, increasing in intervals of 2.5 or 5 mg weekly (maximum dose 20 mg). In addition, 5 mg of folic acid was administered. During the evaluation of the patient, the following laboratory parameters were assessed: blood count, urea, creatinine, electrolytes, and liver enzymes, with no adverse reactions or hepatotoxicity.

The etanercept dose was 25 mg subcutaneously, twice a week. Locally, moisturizers and keratolytic agents were used: urea 20% and 40%, calcipotriol/betamethasone cream, and loratadine 1 tablet daily. The duration of treatment varied depending on the response of each patient, resulting in satisfaction in most cases, where the intensity of skin involvement was evaluated by PASI before and after treatment.

The variables used were age, weight, height, BMI, personal and family pathological history, PASI index at the beginning and end of treatment, and laboratory parameters. Only complete clinical histories were included using the international classification ICD-10 L40, excluding patients with other types of dermatological pathology. Retrospective, observational, descriptive, cross-sectional, correlational, and differential analytical methods were used. The SPSS V26.0 program (IBM Corporation, Armonk, NY) was used for data analysis. The percentages, prevalence, and ranges of the variables were compared using differential analytical statistical tests (*P* < .05).

## 3. Results

A prevalence of 3.33% (94/2820 × 100) of moderate and severe psoriasis was obtained Table 1. The mean age was 52.2 years (range 1–88 years), with a standard deviation of 17.9 years. The percentage of male patients was 71.35% (67) and that of female patients was 27.7% (27), with male patients predominating. Based on height and weight, the BMI was calculated, and the average was 28.13, which, according to the WHO classification table is between the overweight and pre-obesity ranges.

**Table 1:** Prevalence of moderate and severe Psoriasis in the Naval Hospital of Guayaquil 2020–21.

$Prevalence:\frac{94patientswithplaquepsoriasis}{2620peopleattendedattheMilitaryHospital.}=3.58\%.$

Source: Naval Hospital of Guayaquil.

Elaborate by: Benites E, Carrillo E, Heras M.

Among the personal pathological antecedents, 35 patients presented with various pathologies such as hypertension (15), type II diabetes (8), allergic rhinitis (6), hypothyroidism, hepatic steatosis, breast cancer, atopic dermatitis, rheumatoid arthritis, and vitiligo, among others. Regarding family pathological history, only 2 patients reported having relatives with moderate psoriasis.

**Figure 1.**
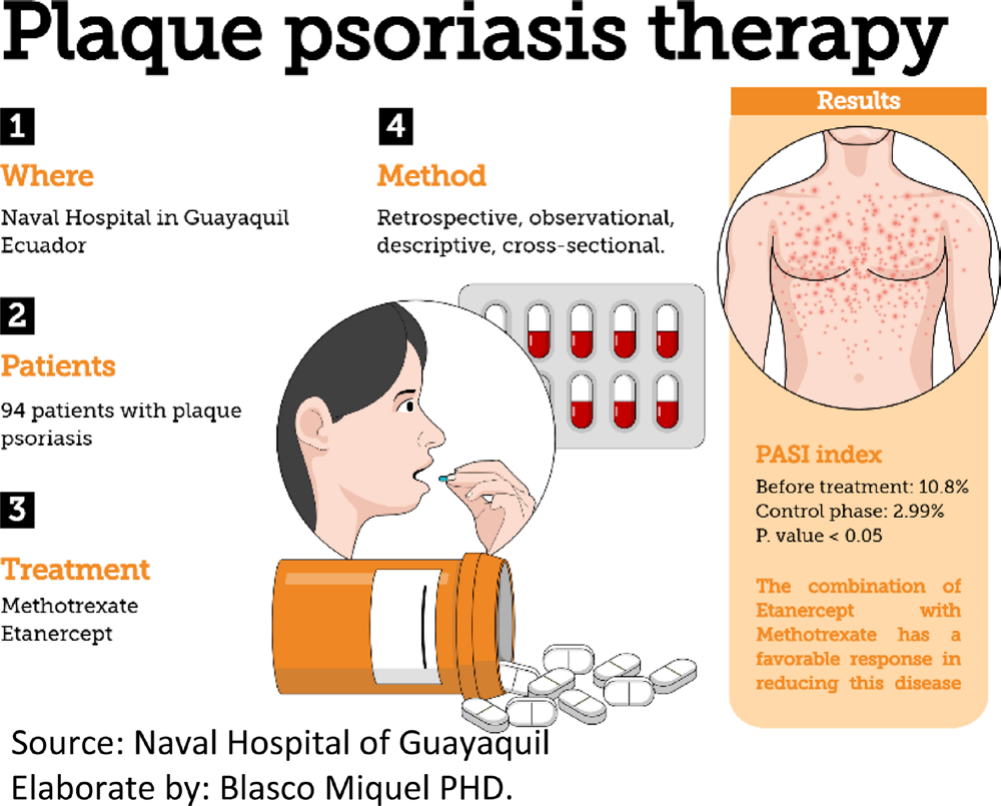
Therapy with Etanercept and Methotrexate in patients with moderate and severe Psoriasis.

The average PASI in the initial stage before treatment was10.8% corresponding to moderate and severe psoriasis and in the control phase of treatment, there was a big significant decrease of 2.99%. (Fig. 1 and Table 2)

**Table 2:** Mean and dispersion of PASI at admission and control in patients with moderate and severe plaque psoriasis at the Dermatological Clinic of the Hospital Naval of Guayaquil 2020–21.

Descriptive Statistic	N	Mínim	Máxim	Half	Standard deviation
PASI UPON ADMISSION (%)	94	.9	33.0	10.827	70.759
PASI TO CONTROL (%)	94	.00	22.30	29.941	341.426

Source: Naval Hospital of Guayaquil.

Elaborate by: Benites E, Carrillo E, Heras M.

PASI = Psoriasis area and severity index.

In Pearson’s correlation in the initial stage, the age/IMC and PASI results were 0.334 and 0.381 > *P* = .05, respectively although most of these patients were young adults with overweight and pre-obesity (Table 3).

**Table 3:** Pearson correlation in moderate and severe psoriasis between age/BMI and PASI at admission in patients of the dermatological clinic of the Hospital Naval in Guayaquil 2020–21.

Correlations		AGE	PASI UPON ADMISSION (%)
** AGE**	Pearson´s Correlation	1	0.101
	Sig. (bilateral)		0.334
** PASI UPON ADM (%)**	Pearson´s Correlation	0.101	1
	Sig. (bilateral)	0.334	
**Correlations**		AGE	PASI UPON ADMISSION (%)
** BMI**	Pearson´s Correlation	1	0.085
	Sig. (bilateral)		0.418
** PASI UPON ADM (%)**	Pearson´s Correlation	0.085	1
	Sig. (bilateral)	0.418	

Source: Naval Hospital of Guayaquil.

Elaborate by: Benites E, Carrillo E, Heras M.

BMI = body mass index, PASI = Psoriasis area and severity index.

Student’s *t* test was used the etanercept and methotrexate treatment in the PASI evolution in the initial phase and at the end of the control phase was lower 0.001 < *P* = .05 demonstrating a highly significant response, previously referred to in the PASI evaluation, where the scaly and erythematous lesions had an excellent response to this type of biological treatment (Table 4).

**Table 4:** Student’s *t* test in moderate and severe Psoriasis between PASI at admission and control of Etanercept and Methotrexate in patients at Hospital Naval of Guayaquil 2020–21.

	Paired sample test	Paired differences		t	Sig. (bilateral)
		Half	Standard Deviation		
Par 1	PASI UPON ADMISSION (%) PASI CONTROL (%)	783.245	657.510	11.549	.000

Source: Naval Hospital of Guayaquil.

Elaborate by: Benites E, Carrillo E, Heras M.

PASI = Psoriasis area and severity index.

In the cross-test of risk estimation, we see that when glycemia values exceed the normal limit oh > 120 mg/dL, it constituted a risk factor in the response to treatment with etanercept (2.231 > Relative risk = 1), (Table 5) and methotrexate (2.008 > Relative risk = 1 (Table 6). Although the majority were overweight, this did not affect the end result of this treatment, showing a significant value of what was expected, which came from with PASI values before and after treatment.

**Table 5:** Ch2 There is a risk in moderate and severe plaque psoriasis when administering Etanercept when Glycemia values are greater than 120 mg/dL of the Hospital Naval in Guayaquil 2020–21?.

Risk estimate	Value	Confidence interval of 95%	
		lower	Higher
Advantage reason for Glycemia (not/yes)	0.385	0.102	1.45
For cohort ETANERCET = Without treatment	0.858	0.715	1.029
For cohort ETANERCET =	2.231	0.699	7.114
With treatment	94		
Valid cases number			

Source: Naval Hospital of Guayaquil.

Elaborate by: Benites E, Carrillo E, Heras M.

**Table 6:** Ch2 There is a risk in moderate and severe plaque psoriasis when administering Methotrexate when Glycemia values are higher than 120 mg/dL of the Hospital Naval in Guayaquil 2020–21.

Risk estimate	Value	Confidence interval of 95%	
		Lower	Higher
Advantage reason for Glycemia (not/yes)	0.461	0.093	2.282
For cohort METHOTREXATE = Without treatment	0.92S	0.805	1.063
For cohort METOTREXATO: With treatment	2.008	0.462	8.717
Valid cases number	94		

Source: Naval Hospital of Guayaquil.

Elaborate by: Benites E, Carrillo E, Heras M.

Limitation: In the hospital, there are no genetic or molecular tests that would have allowed us to compare the type of circulating interleukin, they are active and passive service military patients with a diagnosis of moderate and severe psoriasis that during the time of confinement due to the Covid19 pandemic many were infected, and there was a restriction in the number of patients seen in outpatient consultation; however, some were seen by teleconference as well as post-treatment control.

## 4. Discussion

In the Military Hospital of Guayaquil, drugs and biological therapies topical and oral urea, topical calcipotriol, local betamethasone, loratadine tablets, methotrexate, and etarnecept, were used in 94 patients with a mean age if 52 years among others. Methotrexate was used as a systemic anti-inflammatory therapy, mainly in moderate and severe psoriasis, a doses of 0.2 to 0.4 mg/kg per week. Patients had to be carefully monitored for side effects such as bone marrow suppression or hepatotoxicity^[13]^ Etanercept which is a biologic was administered orally at 50 mg^2^/week subcutaneously for 12 weeks, followed by 50 mg subcutaneously, 1/week, this biologic aids in blocking inflammatory cytokine activity. Analyzing the outcomes with methotrexate and etanercept gave us a highly significant result in the improvement, although most patients were overweight and pre-obese, when evaluating the PASI at the beginning at 10.8% and control at 2.99%, the response to treatment decreased the clinical picture characteristics of moderate and severe psoriasis. This study corroborates a work conducted at the Hospital “Carlos Andrade Marín de Quito, Ecuador from 2010 to 2017 Treatment using infliximab had an improvement of 76.4% (± 21.7), etanercept 65.88%, adalimumab 86.2 (± 13.6) and secukinumab 94.1% (± 5. 3), concluding that biologic therapy in moderate, severe and refractory psoriasis achieves a high percentage of improvement in most patients, regardless of the selection of the biologic drug that is not influenced by the demographic characteristics of the population, but by its availability, without modifying the final clinical response.^[14]^

In the management of comorbidities, the average BMI was 28.13 which corresponds to overweight and pre-obesity, and some are referred to as hypertension, diabetes type2, rheumatoid arthritis, allergic rhinitis, breast cancer and vitiligo. In a study carried out in Mexico on comorbidities in plaque psoriasis, 76.31% of the population was overweight and obese, with a prevalence of 4.3%,^[15]^ which is similar to the prevalence of 3.33% reported in our study.

This type of comorbidity often has implications for the types of drug and biological treatment. In numerous studies, which have been published, the effectiveness of different therapies, has been negatively affected by increased BMI.^[16]^

In recent years, the introduction of new biological agents has demonstrated efficacy in moderate and severe stages, with a secondary literature review highlighting the biologics as the most scientific evidence. Systemic treatments can reduce the severity of symptoms and prevent further damage. Therefore, timely therapy should be initiated to stop the progression of cutaneous symptoms and possibly reduce systemic ones.^[17,18]^

In our study, the treatment with etanercept and methotrexate had a significantly reduced the clinical symptoms of this disease. In a published study of a radiographic efficacy, safety, double-blind, randomized, radiographic clinical trial in 686 patients with active rheumatoid arthritis who were randomized to treatment with etanercept 25 mg (subcutaneously twice weekly) and oral methotrexate (up to 20 mg per week), an improvement in functional disability and delayed radiographic progression was demonstrated in this combination compared to methotrexate or etanercept alone. These findings bring us closer to achieving remission and repair of structural damage in rheumatoid arthritis.^[9]^

It is expected that in Ecuador, the biologics used for the treatment of moderate and severe psoriasis will be included in the basic drug list of the Ministry of Public Health, with endocrine, metabolic, articular bone, and psychosocial factors as comorbidities. For this reason, psoriasis is considered a multisystemic disease and is at risk of presenting complications that may affect the quality of life of these patients at a personal, family, and work levels.

## Additional contribution

The authors are grateful for the collaboration of the Military Hospital in obtaining the database. Ing, César Pincay, Dr Mario Paredes MSc, Blasco Miquel PHD of the Catholic University in Guayaquil, and Dr José Moleón of the Granada University in Spain for helping us in the critical revision of the manuscript, all of whom agreed to be named in this work.

## Author contributions

Author Contributions: Benites E, Carrillo E, Heras M, had full access to the study database and assumed responsibility for the integrity of the data and accuracy of the data analysis.

**Administrative, technical, or material support:** Elizabeth Benites, Esmeralda Carrillo, Martha Heras.

**Analysis and interpretation of the important intellectual content:** Elizabeth Benites, Esmeralda Carrillo, Martha Heras.

**Critical revision of manuscript:** Elizabeth Benites, Esmeralda Carrillo, Martha Heras.

**Formal analysis:** Elizabeth Benites, Esmeralda Carrillo, Martha Heras.

**Investigation:** Elizabeth Benites.

**Manuscript drafting:** Elizabeth Benites, Esmeralda Carrillo.

**Methodology:** Elizabeth Benites.

**Statistical analysis:** Elizabeth Benites.

**Study concept and designs:** Elizabeth Benites, Esmeralda Carrillo.

**Study supervision:** Elizabeth Benites, Esmeralda Carrillo.

## Referenced articles

Emery, P, Breedveld, FC, Hall, S, et al. Comparison of methotrexate monotherapy with a combination of methotrexate and etanercept in active, early, moderate-to-severe rheumatoid arthritis (COMET): a randomized, double-blind, parallel treatment trial. Lancet 2008;372:375–82. http://dx.doi.org/10.1016/S0140-6736(08)61000-4

Zachariae C, Mørk NJ, Reunala T, et al. The combination of etanercept and methotrexate increases the effectiveness of treatment for active psoriasis despite the inadequate effect of methotrexate therapy. Acta Derm Venereol. 2008;88:495–501. http://dx.doi.org/10.2340/00015555-0511

Driessen RJB, van de Kerkhof PCM, de Jong EMGJ. Etanercept combined with methotrexate for high-need psoriasis. Br J Dermatol. 2008;159:460–463. http://dx.doi.org/10.1111/j.1365-2133.2008.08669.x
